# Measuring urban quality and change through the detection of physical attributes of decay

**DOI:** 10.1038/s41598-023-44551-3

**Published:** 2023-10-12

**Authors:** Andrea Vallebueno, Yong Suk Lee

**Affiliations:** 1 Regulation Evaluation and Governance Lab, Stanford University, Stanford, 94350 USA; 2 Keough School of Global Affairs, University of Notre Dame, Notre Dame, 46556 USA

**Keywords:** Socioeconomic scenarios, Sustainability

## Abstract

The quality of the urban environment is crucial for societal well-being. Yet, measuring and tracking the quality of urban environment, their evolution, and spatial disparities is difficult due to the amount of on-the-ground data needed to capture these patterns. The growing availability of street view images presents new prospects in identifying urban features. However, the reliability and consistency of these methods across different locations and time remains largely unexplored. We aim to develop a comprehensive index of urban quality and change at the street segment level using Google Street View (GSV) imagery. We focus on eight object classes that indicate urban decay or contribute to an unsightly urban space, such as potholes, graffiti, garbage, tents, barred or broken windows, discolored or dilapidated façades, weeds, and utility markings. We train an object detection model on a dataset of images from different cities and assess the performance of these urban indices. We evaluate the effectiveness of this method in various urban contexts over time and discuss its potential for urban planning and public policy. We demonstrate the use of these indices in three applications: the Tenderloin in San Francisco, the Doctores and Historic Center neighborhoods in Mexico City, and South Bend, Indiana.

## Introduction

More than two thirds of the world’s population are expected to live in cities by 2050^1^. As the world urbanizes, the quality of the urban physical environment will become increasingly critical to people’s well-being and sustainable development. Accurate measurements of the quality of the urban space are essential to better design and assess policies that aim to address infrastructure and transportation improvements, poverty, and the health and safety of urbanites, as well as the increasing inequality within and across cities.

The measurement of urban quality and quality of life in urban spaces has long been an active area of research in the social sciences literature. Traditionally, urban quality has been quantified through two main approaches. Administrative data has been used to capture sociodemographic and economic characteristics of the urban space such as crime rates, income levels and housing conditions. Another approach has been to use survey data^2,3^ to examine urbanites’ perception and valued attributes of the urban environment^4^. More recently, there have been efforts to use large image datasets to describe the urban space and its socioeconomic qualities. An early set of papers used satellite imagery to quantify urban sprawl^5^, and nighttime light data to directly proxy for economic activity and growth^6^. Since then, the use of satellite imagery has expanded, especially to examine regions with poor economic data^7–11^.

In the machine learning literature, satellite imagery has been used to generate large-scale mappings of poverty, wealth and income^12,13^, and of other socioeconomic outcomes that are highly expensive to monitor in developing countries, such as infrastructure quality^14^. Using a ground-level approach, the neighborhood assessment literature has measured physical characteristics of the urban environment with street view images. Urban assessments have been performed to predict neighborhood safety^15^, estimate the demographic makeup of neighborhoods^16^ and quantify the quality of building façades^17,18^.

The measurement of urban change has largely centered on quantifying urban growth, primarily by examining land use and land cover dynamics^19^ and change in urban infrastructure^20,21^. At the street level, some works have studied changes to the physical urban environment^15,22,23^ but less attention has been devoted to identifying and measuring urban change that captures the deterioration of urban environments over time. As important as urban growth and gentrification may be, we believe that understanding the deterioration of urban space is just as essential to documenting urban growth and revival. Many cities in California such as San Francisco, San Jose, and Los Angeles have seen a surge in homeless population and encampments. Parts of Chicago and Detroit have seen an exodus of residents, and the declining areas of the cities suffer from increased crime, abandoned buildings, and crumbling infrastructure. A key challenge to urban planning and social policy is to correctly identify the scope and extent of urban decay. In other words, the measurement of urban decay is fundamental for urban policy. However, urban policy and governance generally devote more attention to urban improvements rather than deterioration. Moreover, the measurement of urban decay is further complicated by the fact that on the ground measurements of urban environments are often expensive to collect, and can at times be more difficult, and even dangerous, to collect in deteriorating parts of the city.

Our goal in this paper is to introduce a scalable method to measure urban decay at a spatially granular level over time, and to show that not only do spatial disparities exist within cities over time, but that there is also variation in the measurement of urban decay using computer vision across different cities. Our method produces an index of urban quality and change from street view images of cities over time that is focused on the detection of objects that are evocative of urban decay using an accessible object detection model^24^.

We evaluate our method in three contexts: homelessness in the Tenderloin, San Francisco between 2009 and 2021; a set of small-scale housing projects carried out in 2017–2019 in a subset of Mexico City neighborhoods; and the western neighborhoods of South Bend, Indiana in the 2011–2019 period, a part of the city that had been declining for decades but also saw urban revival initiatives. Our key findings are below.

- We create an urban quality metric at the street segment level, enabling the analysis of local spaces, the mapping of urban inequality at high granularity over time and the comparison of urban decay patterns across geographies. By creating a holistic measurement of the physical decay of the urban space from the incidence of ground-level attributes, we can also analyze the spatial and temporal dynamics of specific urban features such as homeless encampments or façade deterioration. This enables the exploration of the drivers behind urban quality gaps across different geographies, the assessment of the evolution of critical and contemporary urban issues and the measurement of the impact of urban projects on the physical environment.
- We make inference on high frequency GSV images and construct panel data at the street segment level. We show the potential of using such data and the caveats based on GSV panorama frequency, panorama overlap, and sample selection (i.e., GSV image availability over time and space), and offer some practical guidance for the application of GSV images to street segment-level panel data.
- Our experiment shows that a general urban quality detection model has the potential to be applicable in different cities and countries. However, caution should be made to assess the performance and accuracy of such models in different cities and across time before being used for planning purposes. We note that the model may need to be fine-tuned or possibly re-trained to make inference in more suburban or rural settings.
- Given the existence of highly different urban profiles across geographies and its effect on the performance of computer vision models that seek to measure urban quality, we argue that creating a method that is based on the aggregation of individual features allows users to better understand the capacity of the model to generalize to new geographies. In this process, we emphasize that reporting the performance metrics for each urban feature in the temporal and spatial context of interest should become standard. Though this increases the cost of developing such models, urban planning and social policy related to urban decay should be solidly grounded on accurate representations of the urban environment.
- Lastly, computer vision models that aim to identify urban quality and change should be concerned about bias that could arise due to skin color and clothing of the people in the street images, or due to other features that correlate with both the presence of the objects of interest to the model and with specific demographic groups. Most street images de-identify facial features due to privacy concerns. However, skin hue and clothing can easily be identified and has the potential to bias urban quality predictions. Masking of the full individual including clothing may be better than pixelizing. We encourage training vision models on imagery from a diverse set of neighborhoods and cities to limit these kind of potential biases.

## Results

To characterize the quality of an urban space we identify eight physical features that are indicative of urban decay or that contribute to an unsightly urban space—broken, boarded or covered windows and doors, garbage, graffiti, discoloring of or cracks on building façades, weeds on pavement, potholes or cracked streets, tents or tarps used as homeless dwellings on the street, and utility markings on pavement. We measure their incidence at the street segment level using an object detection model, and generate an aggregate incidence metric that is representative of the street segment’s urban quality.

**Figure 1:**
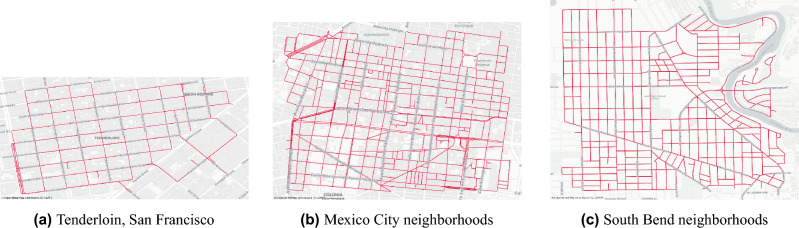
Maps of the streets examined in the three cities. Scales are different across the city maps. Street maps in red illustrate the street segments examined in the paper and were created by authors using OSMnx, a Python package that constructs graphs from OpenStreetMap street network data. Base map in grey is from OpenStreetMap. OpenStreetMap is distributed under the Open Data Commons Open Database License.

We present results from three cities: San Francisco, Mexico City, and South Bend. The neighborhoods were chosen based on a combination of factors, including urban diversity, stages of urban decay, and familiarity with the authors. Figure 1 presents the streets and neighborhoods of each city that we examine in this paper. The primary objective of this paper is to introduce a methodological approach. While we recognize the value of collecting data from a broader range of cities and presenting those results, we believe that the diversity observed in our three case studies sufficiently conveys our central message.

**Table 1:** Test set performance for the YOLOv5 models trained on the consolidated and ex-Mexico datasets.

Model		All classes	Façade	Graffiti	Weed	Garbage	Pothole	Tent	Window	Utility marking
Mission district test set (137 images)										
	Labels	569	68	38	76	215	24	21	93	34
Consolidated	mAP@0.5	0.224	0.005	0.211	0.085	0.245	0.022	0.693	0.478	0.050
	mAP@0.5:0.95	0.113	0.002	0.080	0.030	0.080	0.013	0.393	0.288	0.020
Ex-Mexico	mAP@0.5	0.204	0.009	0.230	0.079	0.205	0.008	0.718	0.355	0.030
	mAP@0.5:0.95	0.105	0.001	0.087	0.033	0.061	0.004	0.402	0.230	0.018
Mexico City neighborhoods test set (121 images)										
	Labels	868	213	155	78	202	48	0	172	0
Consolidated	mAP@0.5	0.220	0.062	0.360	0.293	0.261	0.022	NA	0.324	NA
	mAP@0.5:0.95	0.098	0.018	0.156	0.110	0.094	0.005	NA	0.205	NA
Ex-Mexico	mAP@0.5	0.123	0.031	0.207	0.201	0.107	0.021	NA	0.173	NA
	mAP@0.5:0.95	0.055	0.009	0.114	0.063	0.038	0.006	NA	0.101	NA
South bend neighborhoods test set (130 images)										
	Labels	678	7	0	544	97	29	0	1	0
Consolidated	mAP@0.5	0.036	0.073	NA	0.034	0.072	0.001	NA	0.000	NA
	mAP@0.5:0.95	0.012	0.022	NA	0.011	0.024	0.000	NA	0.000	NA

### Performance across cities

We train two YOLOv5 models, one on the Consolidated and another on the Ex-Mexico object detection datasets (described in the “Methods” section), to understand how relevant the addition of specific training data is when running inference for a new city and to quantify the impact on performance in San Francisco from including training data from new cities with different urban features. Table 1 reports mAP@0.5 and mAP@0.5:0.95 for each class across the three test set locations and the two best-performing models trained on each dataset. For the Mission District test set, the best-performing Consolidated model registers a higher mAP@0.5 for all classes compared to the best-performing Ex-Mexico model. Including the Mexico City training data thus improved inference in this San Francisco neighborhood, mainly driven by the improved detection of windows and potholes which are commonplace in the Mexico City train data. For the Mexico City test set there is a notable gap in performance between the best-performing Consolidated and Ex-Mexico models across nearly all classes. We expect that including imagery from Mexico City allows the model to train on the specific characteristics of class instances in Mexico. The improvement in performance may also be driven by the fact that the Mexico City imagery was sourced from GSV, narrowing the gap between the training and test data.

**Figure 2:**
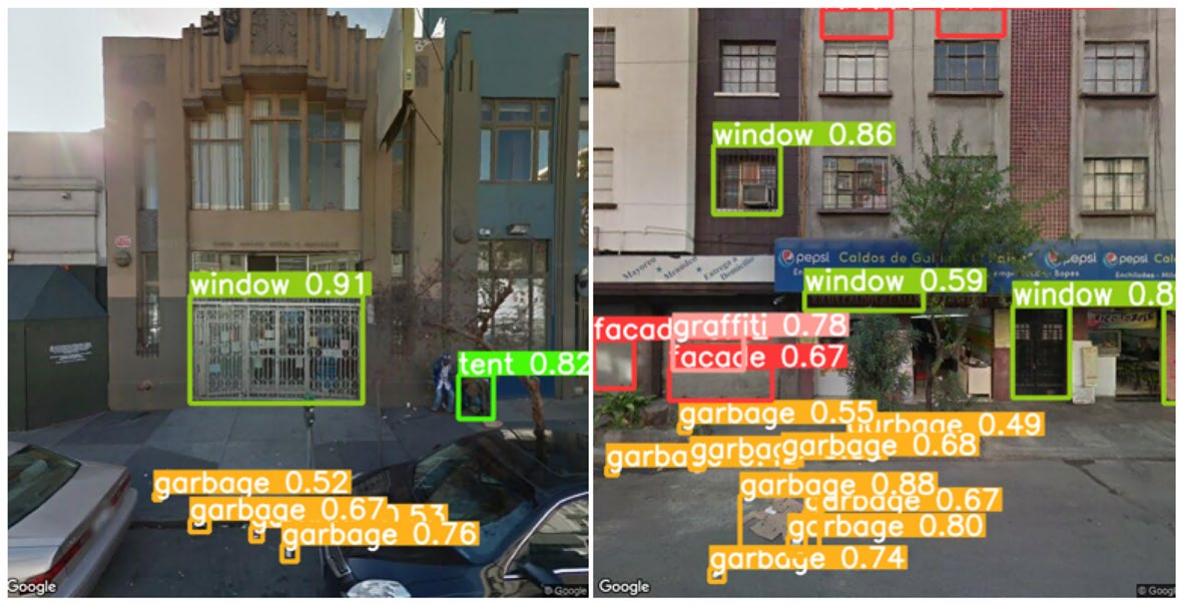
Detection output samples for Tenderloin (left) and Mexico City (right). Detection outputs of Google Street View (GSV) images were generated by the authors using the YOLOv5 repo^24^ as developed by Ultralytics^24^ in the PyTorch framework^25^. On the left, the model correctly identifies a tent instance. On the right, out of the building’s 10 small windows, the model correctly detects a single one as it is the only barred window.

The best-performing Consolidated model registers relatively poor performance in the South Bend test set, resulting from the ubiquity of less urban features in comparison to San Francisco and Mexico City. This is evident in the test set class distribution, which is primarily composed of weeds and, to a lesser extent, garbage, and underscores the limitations of the model when extending it to geographies with a higher prevalence of rural features. The model trained on the Consolidated data was selected to run inference for the below use cases prior to computing test set performance in order to maintain the integrity of the test set. Detection output samples for this model can be found in Fig. 2.

The mean Average Precision (mAP) values provided in Table 1 indicate the challenges we faced in detecting urban attributes; in particular, the table reveals heterogeneous performance across object classes. While classes with well-defined object instances, such as barred windows, performed adequately (mAP@0.5 of 0.478), other classes such as façades, weeds, potholes and utility markings performed poorly. We believe that poorer performance for these classes reflects the difficulty of annotating these specific classes using bounding boxes, rather than the model’s general inability to detect these features, as it is much more difficult to determine what a single unit or object instance should be in this case.

Benchmark performance of the pre-trained YOLOv5s model on the Common Objects in Context dataset (COCO) dataset is a mAP@0.5:0.95 of 0.374. While our reported mAP@0.5:0.95 of 0.113 seems low in comparison, we note that if we evaluate our model on the classes that are less prone to the difficulties stated in the paragraph above (graffiti, garbage, tents and windows) mAP@0.5:0.95 stands at a more robust 0.210. Moreover, it is essential to note that object detection in the context of these smaller objects, such as pieces of garbage and graffiti, is a much more difficult task compared to the larger objects that are typically part of large-scale object detection datasets such as COCO. Other works that have used object detection in this small object setting have reported comparable performance metrics to those evaluated for our model; for example, Ayush et al. register mAP@0.5 of 0.248 in the context of detecting buildings, trucks, passenger vehicles, and other objects from satellite imagery^13^.

### Use cases

#### Tenderloin, San Francisco

**Figure 3:**
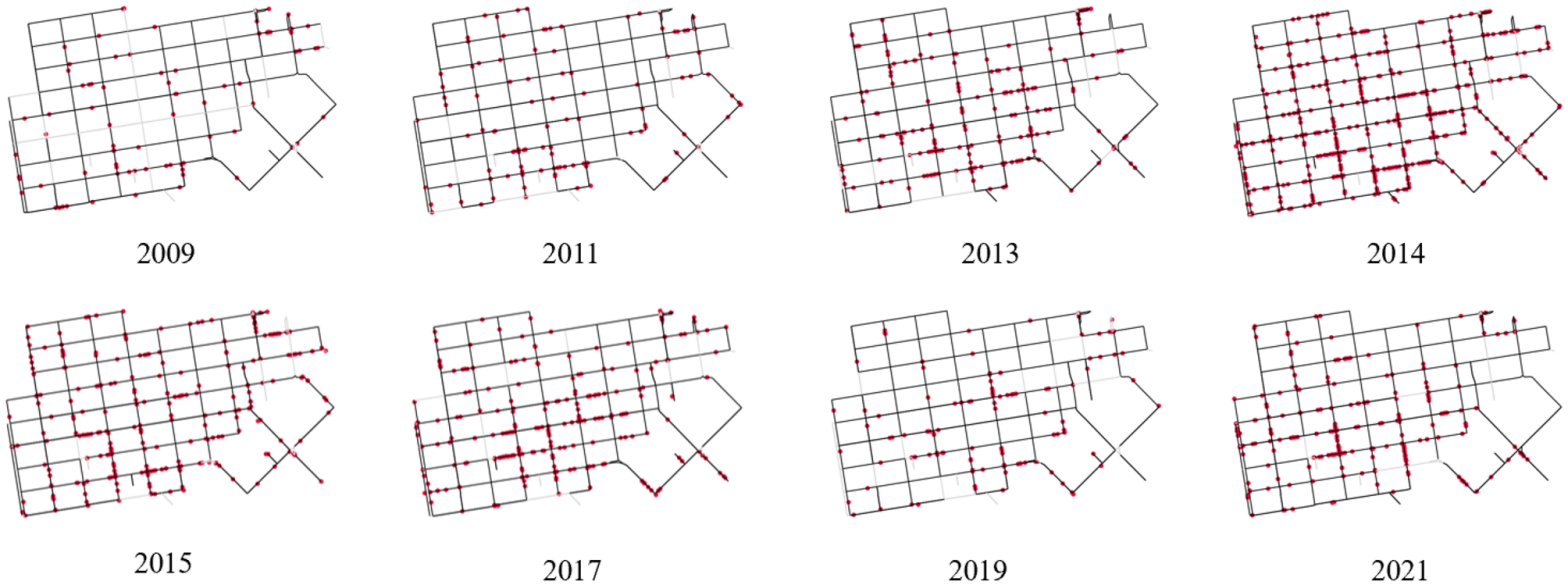
Locations of detected tent instances (red) for all available annual GSV imagery of the street segments in the tenderloin. Street segments with limited or unavailable imagery are colored in light gray. Maps created by authors using OSMnx, a Python package that constructs graphs from OpenStreetMap street network data.

Homelessness has been increasing steadily in the US in the past four to 5 years, reversing the steady decline the US had achieved in the previous decade^26^. The Covid-19 pandemic has created additional challenges for homelessness. Health deterioration from Covid-19 symptoms, business closures, and job losses have affected people’s ability to maintain proper shelter, and at the same time have made it challenging for the government to keep track of homeless populations and implement adequate policy responses. San Francisco, which has one of the highest housing costs in the US, has seen a surge in its homeless population, especially during the pandemic months. The rise of homeless encampments in its neighborhood of the Tenderloin and the surrounding area have not only affected the streetscape but have caused public safety and health concerns prompting calls for public policy intervention and even lawsuits^27^.

For appropriate and timely policy response, the government, NGOs, and the public need reliable statistics on the extent of unsheltered homelessness. However, accurately documenting homelessness is challenging due to the sheer size and numerous streets of metropolises, as well as the personnel and resources required to track homelessness. We believe our urban quality index can provide valuable homelessness statistics that can serve as inputs into designing homeless policy. We illustrate our results in the Tenderloin from 2009–2021 and discuss the benefits and nuances for practical applications.

The Tenderloin is located in downtown San Francisco and is a historic area that has not experienced the level of urban development and growth observed over the past decades in other neighborhoods in San Francisco. Limited gentrification has resulted from several policies, including changes to its zoning laws implemented in the 1980s constraining commercial space to the first floor and limiting building height; protections for Single-room Occupancy (SRO) hotels and historic buildings; and land ownership by NGOs providing affordable housing^28^. Over the past few years, the Tenderloin has seen some new businesses and investments, especially at the neighborhood’s edge, such as the 826 Valencia nonprofit center in 2016 and the development of a mid-rise apartment building at 1066 Market St. in 2020^29^.

For each queried coordinate pair along the Tenderloin’s street segments, all available GSV imagery is downloaded from 2009 to 2021 to explore the change in urban quality that the neighborhood has experienced. As multiple images per coordinate are available from different months throughout each year, we compute urban quality indices for 2011 and 2021 by aggregating the monthly urban quality vector representations of each street segment. From 2011 to 2021, we find that urban quality indices have generally deteriorated across the neighborhood, with higher urban decay in the street segments surrounding Union Square and Civic Center Plaza that capture increased graffiti and some business closures, reflected in the index due to their boarded windows.

Figure 3 visualizes the locations of detected tent instances for all GSV images available at each queried coordinate per year. Segments that have extremely limited or unavailable imagery for a certain year are colored in gray. While these maps are useful to understand the geographic dynamics of homelessness in the area, it is important to note that annual detections are highly dependent upon the frequency of available GSV imagery, which is much higher for specific years. For this reason, the maps are not directly comparable and one must exercise caution in their interpretation. By visualizing these maps we can nonetheless assess the temporal and geographic variation in homelessness in the area. Even taking into account the GSV availability, when we compare 2019 and 2021 we can observe an increase in the detection of homeless camps during the pandemic years, with tents expanding out to new areas for which no tents were detected in 2019. However, Fig. 3 also shows that despite the perception and media report that homelessness expanded in Tenderloin recently, homelessness has been an endemic problem, and may have been worse in the past in 2014. Figure 3 also suggests that aggregation over time will enable one to make a historical assessment of homelessness, or any urban feature, since over time GSV images tend to be available for most street segments.

#### Mexico City neighborhoods

We select a subset of street segments for Mexico City including parts of the Doctores and Historic Center neighborhoods located in the city’s Cuauhtémoc municipality. The imagery is collected for two periods, spanning the time before (2017) and after (2019) the implementation of urban projects related to the 2018 Presupuesto Participativo government program. This program allocates 3% of each municipality’s annual budget to projects proposed by its citizens related to urban infrastructure, construction, crime prevention, and cultural, sporting and recreational activities.

**Figure 4:**
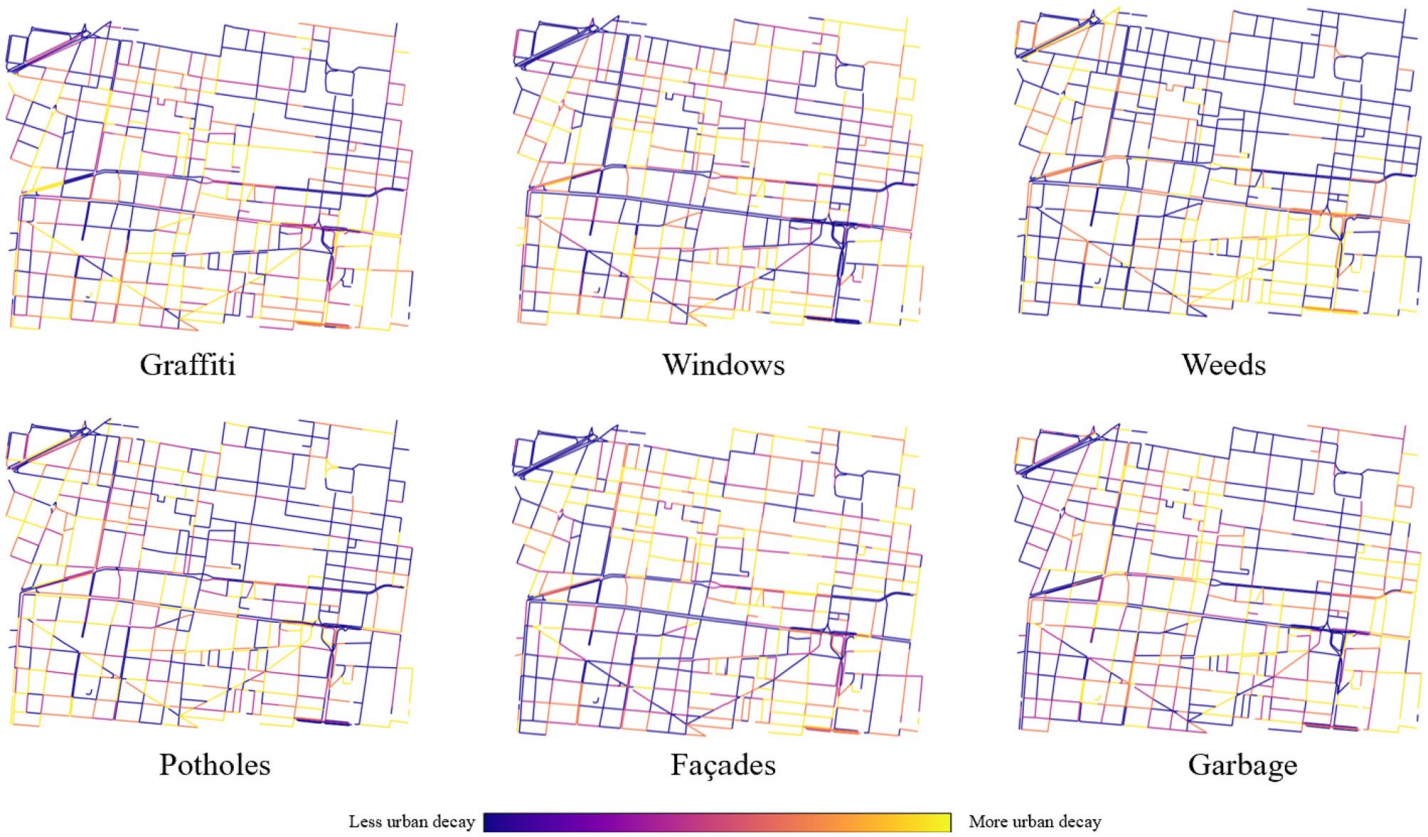
Class-level urban quality indices for the selected street segments in Mexico City for the 2016–2017 period. Tents and utility markings are not plotted due to the absence of their classification. If a street segment became lighter (more yellow) then urban decay (i.e., detection of urban-classes) increased over time. If a street segment became darker (more blue), then decay decreased overtime. Maps were created by authors using OSMnx, a Python package that constructs graphs from OpenStreetMap street network data.

We generate urban quality indices for the 2016–2017 period. A higher quality index is generally observed for street segments along important landmarks, large institutional offices and main avenues, including Mexico City’s main square (Plaza del Zócalo) and a segment of the iconic avenue Paseo de la Reforma. The aggregated urban quality index fails to capture nuances in the distribution of class-level instances. Weeds are rarely observed in the Northern part of the area enclosing the Historic Center and its outskirts, and are more common in the less gentrified spaces of the Doctores neighborhood. In comparison, façade decay is more evenly spread and captures discoloration and paint chipping, as well as cracks in building surfaces and other facade conditions seen across the area. Figure 4 presents the change in individual urban features for Mexico City segments between 2016 and 2017.

**Figure 5:**
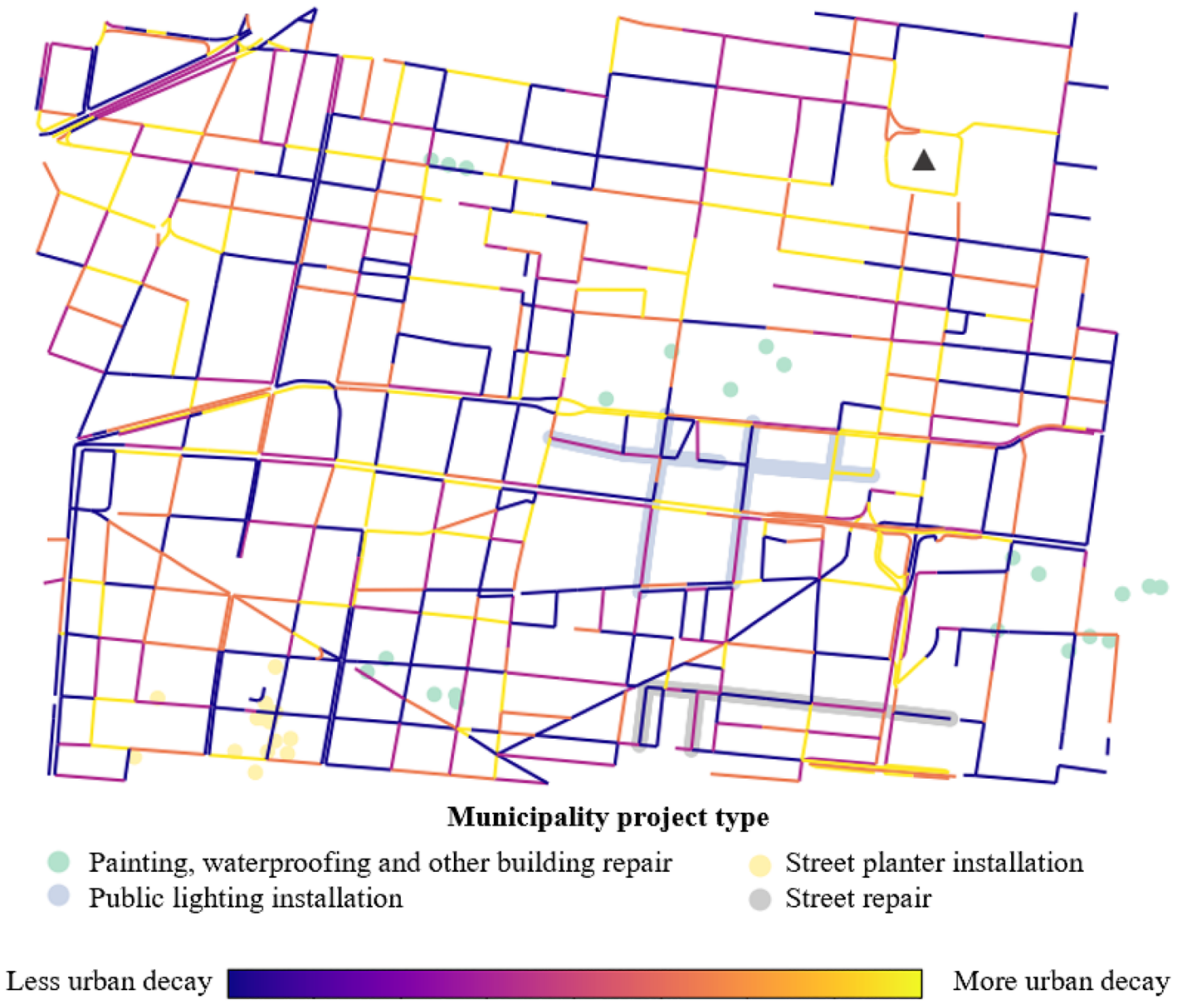
2017–2019 urban quality index change for Mexico City segments. Locations of municipality projects from the Presupuesto Participativo program are colored according to the project type. Plaza del Zócalo, the city’s main square, is marked with a black triangle. If a street segment became lighter (more yellow) then urban decay (i.e., detection of urban-classes) increased over time. If a street segment became darker (more blue), then decay decreased overtime. Map was created by authors using OSMnx, a Python package that constructs graphs from OpenStreetMap street network data.

Figure 5 visualizes the change in the aggregated urban quality index for each street segment from the 2017 period to the 2019 period. In this sense, Fig. 4 offers a more macroscopic perspective on urban quality and Fig. 3 presents a more detailed view of individual urban features and their detections across different time frames. The easy way to understand the urban quality index in these figures is to examine the color variation. If a street segment became lighter (more yellow) then that implies that urban decay (i.e., detection of classes) increased over time. If a street segment became darker (more blue), then our measure of decay decreased overtime. The municipality projects implemented as part of the Presupuesto Participativo program during this same time period in the area are overlaid and colored according to the type of urban feature they impact. The majority of the displayed projects present exterior changes that are visible from GSV images, with the exception of a subset of painting and building repair projects that were performed for building interiors. Some of these projects should have a direct impact on the change in urban quality for a street segment, while others could potentially but not necessarily have an indirect impact on the index. For instance, street planter installations could result in the removal of potholes or weeds along a sidewalk and reduce the measure of its urban decay; however, the sidewalk’s improved aesthetics will not directly result in an improvement in the index. Moreover, even if a project results in a direct change to the urban features captured by the index, it may not affect a sufficient part of the segment to strongly influence a change in its index. With these caveats in mind, we observe that a subset of projects is located on segments that experienced urban quality improvements or relatively less urban quality deterioration (dark blue and purple).

#### Western neighborhoods, South Bend

In our last use case, we examine the performance of our model in a mid-sized city with more suburban and rural features, and where none of our training images came from. In particular, we examine the western neighborhoods of South Bend. South Bend was a vibrant manufacturing city in the early 20th century, but went through a prolonged period of economic decline and population loss during the latter half of the 20th century, and only recently has seen its population rebound. The city took on an initiative called the “1000 houses in 1000 days” between 2013 and 2018 to revitalize the city by tearing down or renovating a large number of vacant and abandoned homes, including the neighborhoods west of downtown, which we focus on.

We captured GSV images for South Bend around 2011 and 2019, and predicted urban quality along available segments. GSV imagery for South Bend is not as readily available, and accordingly street segments with urban quality measures are more sparsely distributed compared to the other two cities. Also, many street segments were not available in both periods, rendering the subset of street segments with an urban change index even smaller.

**Figure 6:**
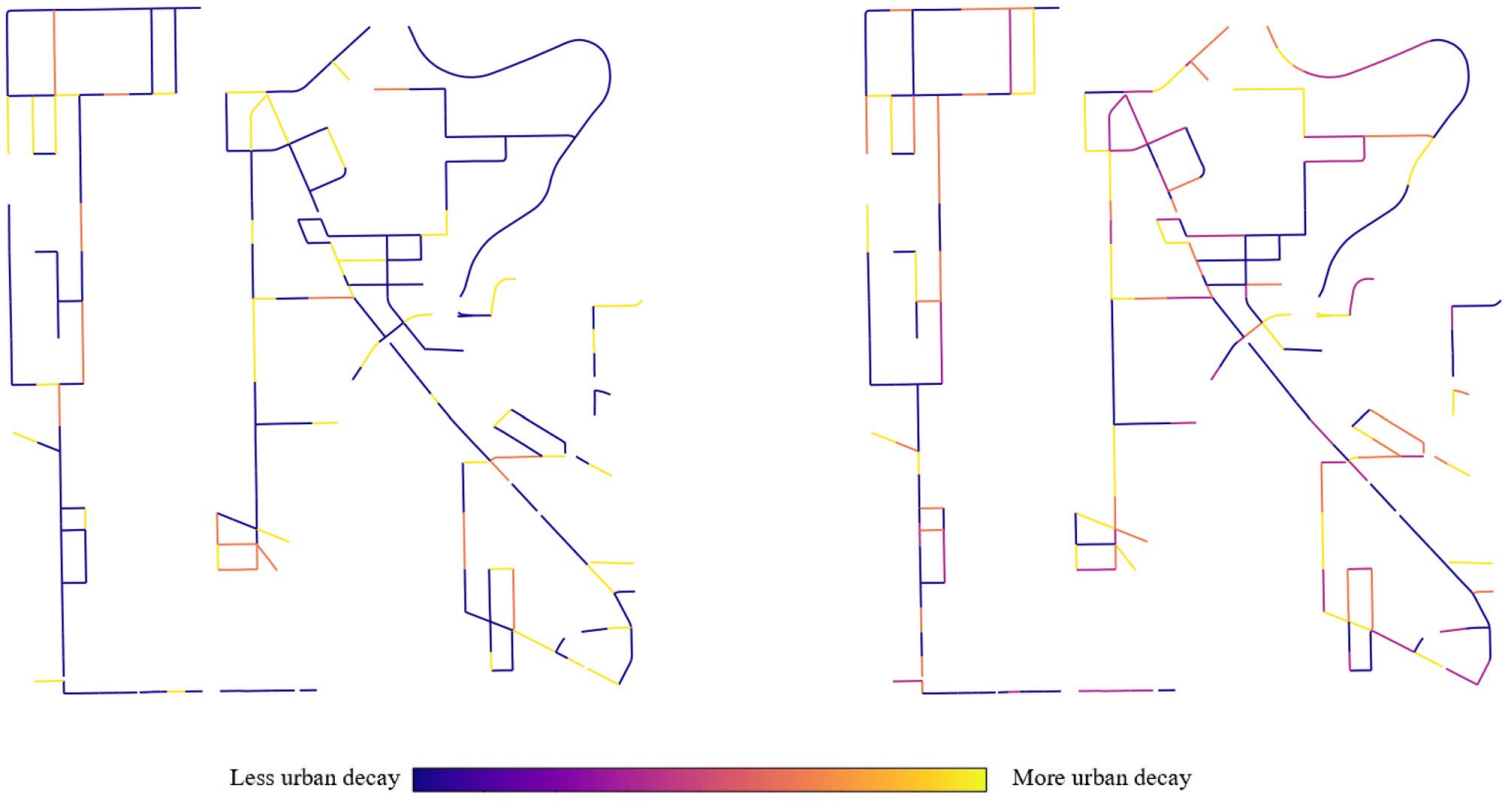
Façade (left) and Weed (right) Incidence Change from 2011 to 2019 in the South Bend Street Segments. We present the two urban features that were most relevant in the context of South Bend. Maps were created by authors using OSMnx, a Python package that constructs graphs from OpenStreetMap street network data.

Though test set performance for South Bend was not as good compared to the more urban San Francisco or Mexico City—the test set included images from a broader area with more rural and suburban streetscapes—the model does a good job of capturing the revitalization that happened in between the time periods. Figure 6 (left) shows the change in façade quality between 2011 and 2019. Most street segments are blue, indicating the highest degree of improvement or smallest amount of urban deterioration. Though façade features are rarely identified in South Bend’s test set, the model does a good job of predicting their change. On the other hand, the prediction results indicate that change in weeds is more varied. This may be due to the challenges the model had in predicting weeds in settings with high levels of vegetation. The South Bend case shows that a general urban quality detection model that works well in cities with dense urban features may need to be augmented or modified when making inference in cities with more suburban or rural settings.

## Discussion

We have taken a computer vision approach to quantifying urban change that has leveraged the detection of urban decay features to build an index of urban quality at the street segment level that can be compared across highly local urban spaces and time periods. The results show that our trained YOLOv5 model is capable of detecting the incidence of these features across different neighborhoods and cities, highlighting the potential of this approach to be scaled in order to track urban quality and change for the entirety of San Francisco, other urban centers across the US and cities in other countries where street view imagery is available. Our visualizations of the constructed indices and their dynamics over time illustrate the value of capturing these physical features of the environment at such a fine-grained level to recognize patterns of neighborhood development and urban inequality that cannot be gleaned using coarser, traditional economic data sources. The generated visualizations serve both as evidence of the patterns of physical urban change that can be sensed by urban dwellers, and as tools to identify and quantify other dynamics of neighborhood growth or decay.

Our experiment shows that a general urban quality detection model has the potential to be applicable in different cities and countries. However, there are considerable disparities in the performance across cities. As our test set performance in Table 1 illustrates, mAP@0.5 is about 22% for both San Francisco and Mexico City, but drops to 3.6% for South Bend. The lower performance for South Bend generally holds for each urban feature that goes into the aggregate index. The disparity reflects the denser urban environments of San Francisco and Mexico City, and the relatively different distribution of urban decay features in South Bend, which is primarily characterized by the presence of weeds. This suggests the need for the training data to be augmented and for the model to be tuned to make inference in more suburban or rural settings.

Overall model performance is impaired by performance on specific object classes; average precision for façades, weeds, potholes and utility markings is much lower than that of other classes such as windows. We believe poorer performance for these classes reflects the difficulty of annotating these specific classes using bounding boxes, as it is much more difficult to determine what a single unit or object instance should be in this case. For future work, we recommend exploring other tasks such as image segmentation to better capture these classes. By annotating all the pixels in an image that belong to a given class, rather than bounding specific class instances as in the case of object detection, image segmentation might be better suited to capturing these features. Second, the detection of small objects is a harder task than the detection of large objects that are typically found in commonly available object detection datasets. We find that our model performance of mAP@0.5 of about 22% is generally in line with that of other works that have used object detection in this small object setting^13^.

Our use cases have illustrated the wide range of urban profiles that neighborhoods can have within and across different cities and countries. For this reason we can expect, and have indeed shown, that notable disparities in the performance of computer vision models that measure urban quality exist across these different locations. For these methods to be rightfully leveraged by urban planning agencies or local governments to serve their local communities, their performance must be monitored such that we ensure they generate faithful representations of the urban environment. Our method, which is based on the aggregation of individual features, allows for the understanding of the model’s capacity to perform in new geographies by evaluating how well the model can detect each specific attribute. For this reason, we emphasize that reporting the performance metrics for each urban feature in the temporal and spatial context of interest should become standard. A second consideration involves the bias that computer vision models can present when looking at urban spaces, particularly that arising due to the skin color and clothing of the people in the street images, or due to the presence of objects that correlate both with urban decay and with the presence of specific demographic groups in a neighborhood. We encourage training these vision models on imagery from a diverse set of neighborhoods and cities to limit these kind of potential biases, and offer further discussion of the relevant considerations to training vision models in urban settings in section.

We would like to underscore that the context, as well as cultural distinctions, play pivotal roles in defining what constitutes decay. For instance, while some may view graffiti as a sign of urban decline, artistic graffiti and murals can indicate a city’s cultural vibrancy and even its revitalization. In our study, we specifically categorized and annotated only graffiti that appeared to be acts of vandalism and intentionally excluded those that had artistic merit. Regarding homelessness, we deem it a significant indicator of urban decay in contemporary cities. Given its increasing prevalence and associated externalities in cities globally, we believe its inclusion in our metrics is important. However, we also recognize the transitory nature of homeless encampments. Our intention in utilizing this measure was to track the shifting locations of these encampments within urban landscapes, and the Google Street View (GSV) platform provides a means to examine such transitions in certain cities. Yet, in cities where Street View imagery isn’t updated regularly, we acknowledge the potential for inaccuracies. This highlights both the advantages and drawbacks of incorporating homelessness as a metric, contingent on the frequency of Street View updates. We do not assume universality in interpreting urban decay and that our indices are not static; they should be adapted to accommodate varying criteria that vary by cities. We believe the strength of our methodology rather lies in its adaptability.

As we noted previously, the model’s diminished efficacy in South Bend, in contrast to its performance in metropolises like San Francisco and Mexico City, illustrates its potential constraints when applied across diverse urban landscapes and the potential limitations concerning generalizability of our model. We wish to emphasize that the model’s applicability is intrinsically linked to the urban setting in question. This methodology would be more relevant in more dense urban regions as opposed to less dense, suburban, or smaller urban locales, such as South Bend. Broadening the scope of training data and the features under consideration when gauging urban quality across varying contexts would increase model performance and potentially generalizability. However, our intent is not to present a one-size-fits-all solution, but rather illustrate the promise of our methodological approach as well as to explore its limitations in different urban contexts. When tailoring our methodology to diverse urban settings, it’s imperative to adjust both the features and their respective training methodologies to resonate with the unique spatial dynamics of the target locale. We hope that our experiments using data primarily from San Francisco, collecting additional training and test sets for new cities (e.g., for Mexico City), and fine-tuning our model in this new context practically illustrates how our approach can be efficiently applied to new urban contexts.

In future work, in addition to further exploring the geographic coverage of our results, we seek to expand upon the notion of urban quality explored in this paper to include features that are not limited to the presence of urban decay, but rather the physical attributes of a space that can improve an urbanite’s positive experience of their surroundings, such as greenery and building aesthetics.

Our study centered on using machine learning to improve our understanding of urban decay and inequality, and by doing so we aimed to contribute to public policy that aims to address urban issues. We focus on physical features of the environment based on street images. We were careful to have each bounding box annotation in the dataset driven only by material objects of the environment, as we did not want the model to inadvertently predict urban decay or homelessness based on the race or ethnicity of the people in the street view images. This can be especially problematic when identifying homelessness and we emphasize that ourselves and other researchers should be careful not to inadvertently confound homelessness or any urban feature with the people on the streets when using street images. One way to reduce this concern has been to blur out people’s faces from images, which GSV already does. However, this may not be enough as people’s clothing and fashion can inadvertently be trained to identify urban features. Also as noted above, skin hue and clothing can still be recognized in blurred images. Masking of the full individual including clothing may be better than blurring or pixelizing. We believe this area warrants further ethical research and we hope this statement will alert other researchers to be mindful when using computer vision even in studies that focus on the physical space.

Our work aligns with the overarching trend identified by Biljecki and Ito^30^, where street view imagery is leveraged for urban analytics. Like some of the studies they reviewed, we employ computer vision to identify and analyze urban features. While many studies focus on specific cities or regions, our research spans multiple cities, each with its unique urban dynamics, providing a broader perspective on urban quality, and our dataset uses street view images from various sources, ensuring a rich and diverse representation of urban features. Our comprehensive urban quality index, which is a synthesis of various detected features, provides a holistic view of urban quality, distinguishing our approach from studies that focus on individual features. Furthermore, our study not only analyzes urban quality at a single point in time but also investigates changes over time, providing insights into urban evolution and development. We believe our choice of the YOLOv5 model and its subsequent adaptation for our specific dataset sets our work apart in terms of methodology and approach, and allows for convenient replicability and adaptability to different contexts. Beyond mere object detection, our research delves into the implications of detected features, bridging urban analytics with urban planning and sociology, and our model’s emphasis on real-time processing positions our study as a precursor to potential real-world applications in urban monitoring and planning.

In conclusion, while our study builds upon the foundational work in urban analysis using street view imagery, we introduce novel methodologies, broader geographical coverage, and deeper interdisciplinary insights, underlining its originality and significance in the field.

## Methods

### Urban features

To build the object detection dataset, we manually collected 1012 images of the streets of San Francisco and annotated each object instance with a bounding box and class label using Roboflow^31^. RoboFlow is an online platform that simplifies and streamlines the processing and preparing of image datasets for computer vision applications. We used RoboFlow to annotate object instances within our images with bounding boxes and class labels. This provided us with a consistent and efficient mechanism for annotation, ensuring that the data was prepared accurately for our model training. We complement this dataset with the STORM graffiti/tagging detection dataset^32^ comprising images of graffiti captured in Athens. It contains images that are rich in urban features, making it suitable for our study on urban quality. Our motivation for using the STORM dataset to augment our training data was to enhance our model’s capacity to generalize to new urban spaces by training on a broader representation of urban environments and on physical variations of the urban features we aim to detect. The complete STORM dataset consists of 1022 images; however, we utilized a random subset of 398 images to incorporate to our dataset for training. This was driven by the fact that only graffiti/tagging objects are annotated in this dataset, rather than the full set of urban features that we seek to detect with our trained model. Since including images with positive but un-annotated examples of urban features would negatively impact model performance, we were required to manually annotate each image using RoboFlow to ensure that the remaining urban features (façade conditions, potholes, garbage, and others) are correctly identified. Due to capacity constraints, we opted for annotating a random subset rather than the complete set of 1022 images. We also collect and annotate 283 Google Street View (GSV) images sourced from San Francisco, Oakland and Los Angeles to specifically capture instances of tents and tarps, due to their lower frequency in the manually collected images, and 200 GSV images from Mexico City.

The collected imagery was consolidated to generate two separate datasets. In order to explore and compare model performance to unseen cities from the US and other countries with potentially different urban features, we create a dataset (“Consolidated”) including all of the collected imagery, and a separate dataset (“Ex-Mexico”) excluding the Mexico City GSV images. In each case, a validation split was created by randomly selecting 300 and 250 images, respectively, from the train set, excluding imagery from the STORM graffiti dataset. A description of the datasets is presented in Table 3 in the Appendix.

We generate three test sets from GSV imagery to evaluate model performance in each location. As a subset of the train sets includes images from Tenderloin, we evaluate model performance in this case by collecting test images from the nearby neighborhood of Mission District. In the case of the Mexico City and South Bend neighborhoods, there is no overlap between train and testing imagery. The final test set comprises 137, 121 and 130 images for Mission District, the Mexico City neighborhoods and South Bend, respectively.

Recognizing the intricacy of annotating urban features, which often hinge on the context and nuances of each image, we opted to undertake the annotation ourselves. This allowed us to maintain uniformity and consistency throughout the process. Over the span of nearly a month, we individually annotated batches of images. Following this, we collaboratively reviewed and discussed the more ambiguous cases to reach a consensus on the optimal annotation approach for such features. This collaborative and iterative process bolstered our confidence in the accuracy and consistency of our annotations. In total, we annotated over 1800 images personally across San Francisco, Oakland, San Jose, Los Angeles, Mexico City, and South Bend. The expanded descriptions are provided in the “Methods” section.

### Street view imagery

**Figure 7:**
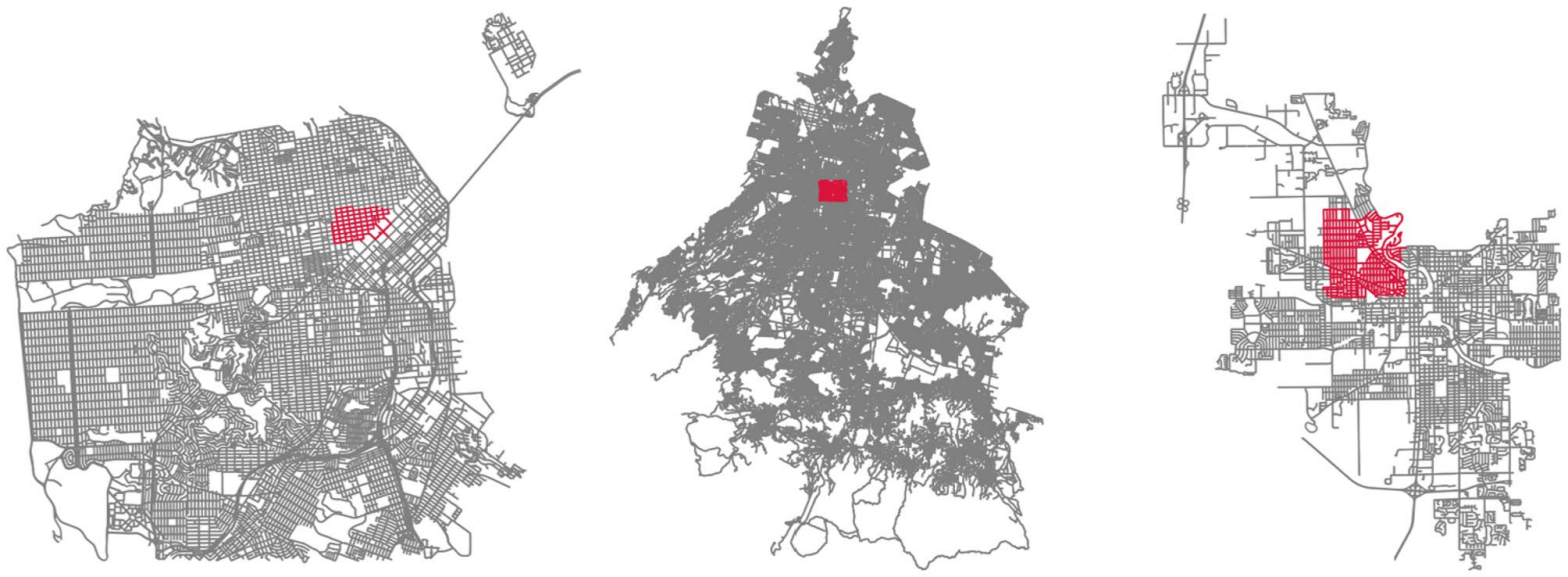
Subset of street segments (red) selected for each of the locations in San Francisco, Mexico City and South Bend. Maps were created by authors using OSMnx, a Python package that constructs graphs from OpenStreetMap street network data.

We generate urban quality and change indices for three locations: Tenderloin, San Francisco; a subset of the Historic Center and Doctores neighborhoods in Mexico City; and the western neighborhoods of South Bend, Indiana. Figure 7 illustrates the subset of street segments selected within each location. Further discussion on neighborhood selection can be found in the “Results” section.

The street imagery is collected from the Google Static Street View API^33^. For each neighborhood, we specify a rectangular area defined by two latitude and longitude pairs from which to generate a graph of the area’s street network. Images are captured at several coordinates along each street segment in the network such that they face the street’s buildings at a right angle on each side, and are downloaded at a 640 × 640 resolution.

The images are downloaded for specific time periods. For Tenderloin we download all available historic panoramas for each queried coordinate pair from Jan-09 to Jul-21, totaling 57,695 images. For the Mexico City and South Bend neighborhoods, we query the images such that at each coordinate pair the panorama with the timestamp closest to a specified date is selected. We also set a date range for each query and do not download panoramas unless their timestamp falls within this range. For the Mexico City neighborhoods, we download images from two time periods: from Mar-16 to Aug-17 with an optimal timestamp of Aug-17 (19,315 images); and from Mar-19 to Mar-20 with an optimal timestamp of Mar-19 (20,342 images). For the South Bend neighborhood, we also download images from two time periods: from Jan-18 to Dec-19 with an optimal timestamp of Aug-19 (10,141 images); and from Jan-09 to Dec-12 with an optimal timestamp of Oct-11 (6,891 images).

The term “optimal timestamp” refers to the ideal date we would like to collect GSV imagery from. Each time we collect imagery for a location, we define a timeframe and an optimal timestamp: we collect any available image that has a timestamp falling within that timeframe—and otherwise declare that the segment has missing imagery –, and, if there are multiple images available, we collect the image that has a timestamp closest to the “optimal timestamp”. As an example, in the Mexico City case for 2017 we wished to collect the latest imagery available before the urban projects were implemented so that we could capture urban change prior to and after the projects were completed. For this reason, we query the GSV imagery from Mar-16 to Aug-17, providing a wide enough timeframe for imagery to be available for the segments, and choose Aug-17 as the optimal timestamp so that available images near the end of the timeframe are prioritized in our collection.

As such, the downloading procedure varied due to differences in data availability across different timeframes, and according to the specific use case for each location. For example, in Mexico City we collect imagery from 2017 and 2019, 2 years with relatively higher availability of GSV imagery compared to other years, to contextualize our 2017–2019 urban change indices in the setting of the urban projects implemented in relation to a 2018 local government program. In the case of South Bend, GSV imagery is updated much less frequently compared to Mexico City and South Bend, and so we examine urban change across a much wider time period by collecting images from 2011 and 2019.

**Figure 8:**
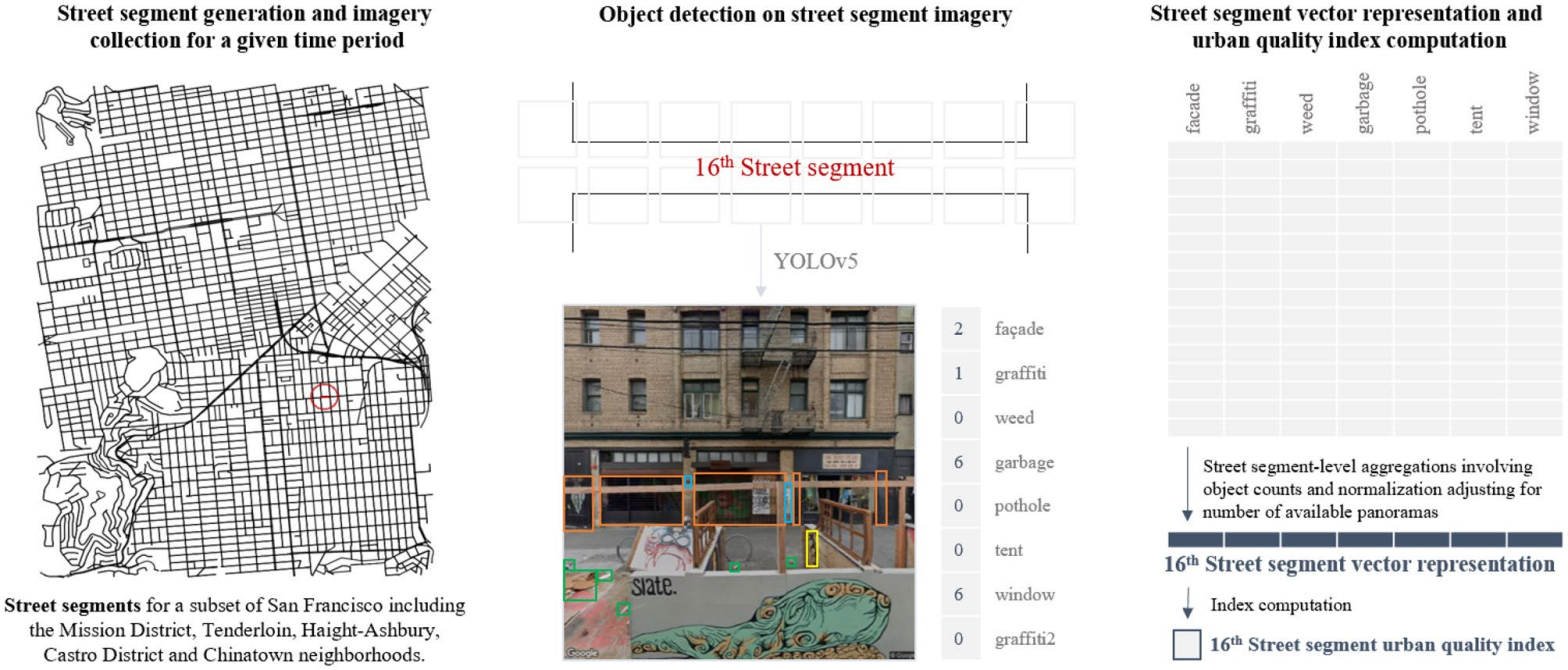
Overview of the proposed pipeline using a subset of San Francisco. GSV imagery for each street segment is queried at 5 m intervals on each side of the street. The object detection model is run on the collected imagery and a vector representation of the street segment is generated by aggregating the image-level outputs of the detection model. Maps were created by authors using OSMnx, a Python package that constructs graphs from OpenStreetMap street network data. Detection outputs of Google Street View (GSV) images were generated by the authors using the YOLOv5 repo^24^ as developed by Ultralytics^24^ in the PyTorch framework^25^.

### Street segment generation and image collection

Street segments for the three neighborhoods were generated using OSMnx^34^, a Python package that constructs graphs from OpenStreetMap street network data. To collect the imagery for the street segments we run an algorithm that traverses each segment at 5 m intervals. At each step, it queries for GSV panoramas available for that latitude and longitude pair, and returns the panorama with the timestamp closest to the selected optimal date within the specified date range. We download two images for each panorama corresponding to the views on each side of the street at a 90$^{\circ }$ angle.

Although the imagery for a street segment is queried at 5 m intervals, the GSV API returns the closest panorama available to a location. Thus, it may be that the collected imagery for a street segment includes images with some overlap in their view of the street or that parts of the street segment are not captured in the available panoramas. The frequency of available panoramas is generally irregular, such that main streets tend to have panoramas at more frequent intervals compared to less active or residential streets. We adjust for this when generating the urban indices for each street segment.

### Object detection

We train a YOLOv5 model with a CSP backbone^35^ as developed by Ultralytics^24^ in the PyTorch framework^25^. While the model architecture is very similar to that of YOLOv4^36^, the YOLOv5 framework incorporates several procedures that facilitate and improve training. These include mosaic augmentation (introduced in YOLOv3^37^) and automatic learning of bounding box anchors, which trains anchors to fit the bounding box size and distribution observed in the training set^38^. hile there are several prominent models available for object detection, the choice of YOLOv5 was motivated by its performance, real-time processing capability, architectural improvements, and adaptability to our specific research needs. In particular, we opted for the YOLOv5 model—the most recent release of the YOLO model at time of writing—for two reasons. First, the YOLO model’s architecture is designed for real-time object detection and processes images in a single pass, which makes it faster than other two-stage detectors like R-CNNs. This capability was particularly beneficial for our study as we aimed to analyze large amounts of street imagery, allowing for quick inference of urban environments. Second, we expected the use of contextual information in the YOLO model to be helpful for our detection task due to the consistent visual structure of the street images—all images were collected at a 90° angle to the direction of the street such that they fully face the sidewalks and buildings—and due to the fact that some of our object classes (e.g., pieces of garbage) are quite small.

Training is performed using a single Tesla P100 GPU. Due to the small size of the object detection dataset, we train the small and medium-sized YOLOv5 models to avoid overfitting, and perform transfer learning to leverage pre-trained weights on the COCO dataset. We use the hyperparameters that are optimized for performance on the COCO dataset as a baseline, including a learning rate of 0.01, an Intersection Over Union (IoU) training threshold of 0.20 and several forms of data augmentation. We experiment with re-training the model end-to-end, and with training while freezing its backbone and neck components. Each experiment is run a single time, for up to 100 epochs. An overview of the explored hyperparameters can be found in Table 2. Model performance is evaluated using standard metrics in object detection, including mean average precision (mAP) at the 0.5 IoU threshold (mAP@0.5) and mAP over different thresholds (mAP@0.5:0.95). The best-performing model trained on each of the two datasets is selected as that reporting the highest mAP@0.5 across all classes. Mean average precision (mAP) is a standard metric used to measure the performance of object detection models, and is computed by calculating the average precision of each class and taking the mean over these values. In the object detection setting, the average precision of class i, $AP_i(t)$, is a function of a threshold t which is used to determine whether the bounding box prediction for an object is correct. Specifically, if the Intersection over Union (“IoU”)—the area of the intersection divided by the area of the union of two geometries—of a predicted bounding box and an object annotation is larger or equal to t, the predicted bounding box is defined as a true positive. If N is the number of classes in an object detection task, then the mAP is computed as $mAP@t= \frac{1}{N} \sum _{n=1}^{N} AP_i(t)$. As model performance varies depending on the IoU threshold, more robust model evaluation is conducted by considering mAP across multiple IoU thresholds. In fact, the Common Objects in Context dataset (COCO), one of the most commonly used large-scale object detection datasets, characterizes model performance by computing mAP at an IoU threshold of 0.5 (mAP@0.5) and by computing the average precision over multiple IoU thresholds ranging from 0.5 to 0.95 with a 0.05 step size (mAP@0.5:0.95), in addition to using other performance metrics. These are the two metrics that we use to benchmark model performance in our work.

**Table 2:** YOLOv5 model explored hyperparameters.

Hyperparameter	Values	Selected value
Batch size	2, 4, 16, 32	4
Frozen layers	0, 10, 24	0
Pretrained weights	Yes, No	Yes
Learning rate	1e−1, 1e−2, 1e−3,	2e−2
	1e−4, 2e−2, 5e−2	
Model size	YOLOv5s, YOLOv5m	YOLOv5m
Optimizer	SGD, Adam	SGD

YOLOv5s is the smallest variant of the YOLOv5 series. It has fewer parameters compared to the other variants. Hence, it is faster but sacrifices a bit of accuracy. YOLOv5m is a medium-sized model, having more parameters than YOLOv5s.

### Street segment vector representations and urban quality index computation

We use the best-performing YOLOv5 model trained on the Consolidated data to run inference on the GSV imagery, which outputs a set of object detections on the images belonging to each street segment. As per^13^, vector representations of each segment are created by summarizing the detections for each class using four aggregation types: object count, object count weighted by detection confidence, object count weighted by bounding box image coverage, and object count weighted by both bounding box image coverage and by detection confidence. For the results, we generate vector representations using the object count aggregation with a minimum confidence level of 50%. In addition to being most intuitive, we found this aggregation to be less susceptible to differences in each panorama’s street view, due to closeness to buildings or view obstruction by large objects such as buses.

We also explore two normalization methods to ensure that the generated vectors for each street segment are comparable: adjusted street length and number of captured panoramas. Due to the irregularity of GSV panorama frequency along streets, vector normalization using street length adjusted for missing panoramas results in incomparable vectors, such that segments with higher panorama frequency tend to have higher object incidence. For this reason, we normalize vectors by the number of available panoramas for each street segment. This modifies the interpretation of the vector elements from one of average number of objects seen per meter for the street segment, to one of number of objects seen in an average view of the street segment.

The urban quality index for a street segment is computed by summing the elements of its vector representation, or by extracting the value of one of its elements to capture the object incidence of a specific class. Urban quality changes in time are calculated as the absolute difference between the logarithm of the urban quality index from each time period. Figure 8 presents an overview of the entire methodological pipeline.

In addition to the urban quality index we use in this paper we experimented with other indices to ensure robustness in our findings. In addressing the variability in the number of detected objects, we employed normalization, averaging the counts across different elements to account for potential discrepancies in the amount of available imagery for a segment. Additionally, we formulated weighted averages, recognizing that different elements have varying impacts on the urban quality index. However, after thorough consideration, we chose the most straightforward metric for several reasons. First, our primary objective was not to derive an absolute index but rather one that effectively captures temporal changes across cities. Utilizing logarithms minimizes the distinction between using the sum or average, as the log predominantly captures relative percentage changes. Second, the significance of the individual elements is likely to vary between cities, a phenomenon evident in our application to three distinct cities. Implementing city-specific weights could compromise the index’s cross-city comparability, leading us to choose the non-weighted version. Lastly, it’s worth noting that our index is not posited as the ultimate measure of urban quality. We acknowledge that there are numerous dimensions to urban quality, encompassing both decay and renewal. Our primary goal and contribution lie in establishing a consistent, systematic method for constructing an urban quality index using widely available street view imagery that can be tailored to specific cities depending on their urban composition and local needs. This index is designed to capture variations both over time and between cities. While the optimal metric may differ based on context, we believe that our paper offers a valuable observational methodology as a foundation for future work.

## Data Availability

This study uses data manually collected by the authors, Public STORM graffiti/tagging dataset, and Google Street Views. Each dataset and its availability is described below. Replication codes and instructions are publicly available online at https://doi.org/10.5281/zenodo.8373112.

## Appendix

### Urban features

Our Urban Features dataset is the object detection dataset used to train the “Consolidated” and “Ex-Mexico” YOLOv5 detection models. It is composed of the following datasets:

- Public STORM graffiti/tagging dataset by^32^. The complete imagery and graffiti annotations for this dataset are available online at https://doi.org/10.5281/zenodo.3238357. As discussed in the paper, we use a subset of 398 images and add the annotations for the remaining classes using Roboflow^31^.
- 1012 manually captured images of San Francisco streets annotated using Roboflow. We cannot share this dataset due to privacy concerns, given the visibility of passerby faces in the images.
- Google Street View (GSV) imagery of San Francisco, Oakland, Los Angeles and Mexico City, annotated using Roboflow. Due to proprietary reasons as per the Google Street View API’s Terms of Service, we are unable to share this dataset.

Table 3 presents the distribution of class labels and number of images in the Urban Features dataset.

**Table 3:** Class label distribution and number of images in the object detection training and validation datasets.

Dataset	Imagery	All classes	Façade	Graffiti	Weed	Garbage	Pothole	Tent	Window	Utility marking	Images
Ex-Mexico	San Francisco	12,405	3676	1316	2280	2158	670	199	686	1420	1012
	STORM Dataset	2890	1028	1234	230	159	129	0	87	23	398
	California GSV	3021	214	186	228	1341	83	760	138	71	283
		18,316	4918	2736	2738	3658	882	959	911	1514	1693
Consolidated	Mexico City GSV	1829	576	295	27	563	105	2	260	1	200
		20,145	5494	3031	2765	4221	987	961	1171	1515	1893

### Street view

The Street View dataset includes the GSV imagery collected for each of the three locations: Tenderloin, Mexico City and South Bend. This is the imagery used to run inference using the Consolidated object detection model. Due to the proprietary nature of this dataset, we are prohibited from sharing it.
